# Social influences on survival and reproduction: Insights from a long‐term study of wild baboons

**DOI:** 10.1111/1365-2656.12887

**Published:** 2018-08-21

**Authors:** Susan C. Alberts

**Affiliations:** ^1^ Departments of Biology and Evolutionary Anthropology Duke University Durham North Carolina; ^2^ Institute of Primate Research National Museums of Kenya Karen Nairobi Kenya

**Keywords:** baboons, early life effects, environmental effects, long‐term study, primates, reproduction, social behavior, survival

## Abstract

1. For social species, the environment has two components: physical and social. The social environment modifies the individual's interaction with the physical environment, and the physical environment may in turn impact individuals’ social relationships. This interplay can generate considerable variation among individuals in survival and reproduction. Here, I synthesize more than four decades of research on the baboons of the Amboseli basin in southern Kenya to illustrate how social and physical environments interact to affect reproduction and survival.
2. For immature baboons, social behaviour can both mitigate and exacerbate the challenge of survival. Only *c*. 50% of live‐born females and *c*. 44% of live‐born males reach the median age of first reproduction. Variation in pre‐adult survival, growth and development is associated with multiple aspects of the social environment. For instance, conspecifics provide direct care and are a major source of social knowledge about food and the environment, but conspecifics can also represent a direct threat to survival through infanticide.
3. In adulthood, both competition (within and between social groups) and cooperative affiliation (i.e. collective action and/or the exchange of social resources such as grooming) are prominent features of baboon social life and have important consequences for reproduction and survival. For instance, adult females with higher social dominance ranks have accelerated reproduction, and adult females that engage in more frequent affiliative social interactions have higher survival throughout adulthood.
4. The early life environment also has important consequences for adult reproduction and survival, as in a number of other bird and mammal species. In seasonal breeders, early life effects often apply to entire cohorts; in contrast, in nonseasonal and highly social species such as baboons, early life effects are more individual‐specific, stemming from considerable variation not only in the early physical environment (even if they are born in the same year) but also in the particulars of their social environment.

## 1 INTRODUCTION

If the essence of life is to capture energy from the environment and convert it into more organisms (Ellison, 2017), then the essence of social life is to bias the distribution and conversion of this energy via social interactions. In other words, every animal's survival and reproduction are determined by how it interacts with its environment, and for social species, this includes both the physical and the social environments. The physical environment includes both abiotic and biotic components (weather, food and predators), and the social environment is created by the behaviour of the individual and its conspecifics. The modulation of resource distribution by social behaviour—particularly competitive and cooperative behaviour—can generate considerable variation in individual fitness. Thus, social behaviour, in addressing the challenges of the physical environment, creates new challenges of its own.

How do the challenges and opportunities of the physical and social environments unfold over the course of an animal's life, and how do animals meet them? Here, I synthesize more than four decades of long‐term research on the ecology and social behaviour of baboons of the Amboseli basin to illustrate some answers to this question. I begin with a description of the setting of the long‐term Amboseli Baboon Research Project and with background on baboon ecology. I then focus on four phases of the baboon life history to illustrate the interplay of social and physical environments in survival and reproduction.

First, I examine survival during the infant and juvenile period, to illustrate how social behaviour can both mitigate and exacerbate the challenge of survival for young baboons. Social interactions are essential for young baboons as they learn to forage, and social interactions can also mitigate disease risk. However, immature baboons can also die from conspecific attacks. Thus, infant and juvenile baboons must navigate a complex set of social interactions to successfully run the Darwinian gauntlet.

Second, I examine growth and development. Here, social and environmental influences show particularly complex interactions. For example, demography affects maturation rates, but in a sex‐specific manner: living in groups with more adult females accelerates male maturation but slows female maturation. This example and others in this section highlight the dual nature of conspecific relationships, which present both challenges and opportunities.

Third, I examine social interactions in adulthood. Affiliative interactions are strongly linked to adult survival in baboons: Females with more frequent affiliative interactions have higher survival. However, competitive behaviour is also a central feature of baboon social life. Two modes of competition—within‐group competition and between‐group competition—shape baboon social life and again highlight the dual challenges and opportunities represented by social relationships.

Fourth, I examine the “long reach” of early life and describe the ways in which the early life environment affects fitness outcomes in baboons. I develop two themes in this section. The first is the potential importance of social effects in early life. In most animals for which early life effects have been studied, the environmental variables of interest (usually rainfall or population density)—are linked primarily to nutrition and energy intake. In contrast, in humans and perhaps other highly social species such as baboons, the social environment appears to be important, perhaps independent of their consequences for nutrition and energy intake. Understanding how and why social environments in early life affect development and adult survival will be very important for understanding the evolution of complex societies, including our own species. The second theme in this section is the difference between cohort effects—aggregate environmental features that all individuals in a population or social group experience if they are born at the same time—and individual effects. I argue that individual effects—arising at least partly from individual‐specific social circumstances—are a much more important feature in the lives of nonseasonal, highly social species than are cohort effects.

I conclude with a short discussion of the importance of long‐term studies, which are essential for understanding the effects on fitness of both physical and social environments for three reasons (Clutton‐Brock & Sheldon, 2010). First, both physical and social environments change over time. Thus, studying any trait during a short time window will reveal only a small part of the reaction norm for that trait, because short‐term studies capture only a small part of environmental and trait variation. Second, questions about both development and senescence require many years of data, particularly for animals with long life spans. Third, events that occur at one stage of the life span can have profound effects on behaviour, reproduction and survival at other stages. These points resonate throughout this synthesis, which aims to highlight the value of longitudinal, individual‐based data from natural animal populations for understanding both social and environmental influences on fitness outcomes.

## 2 SETTING OF THE LONG‐TERM STUDY

**Figure 1:**
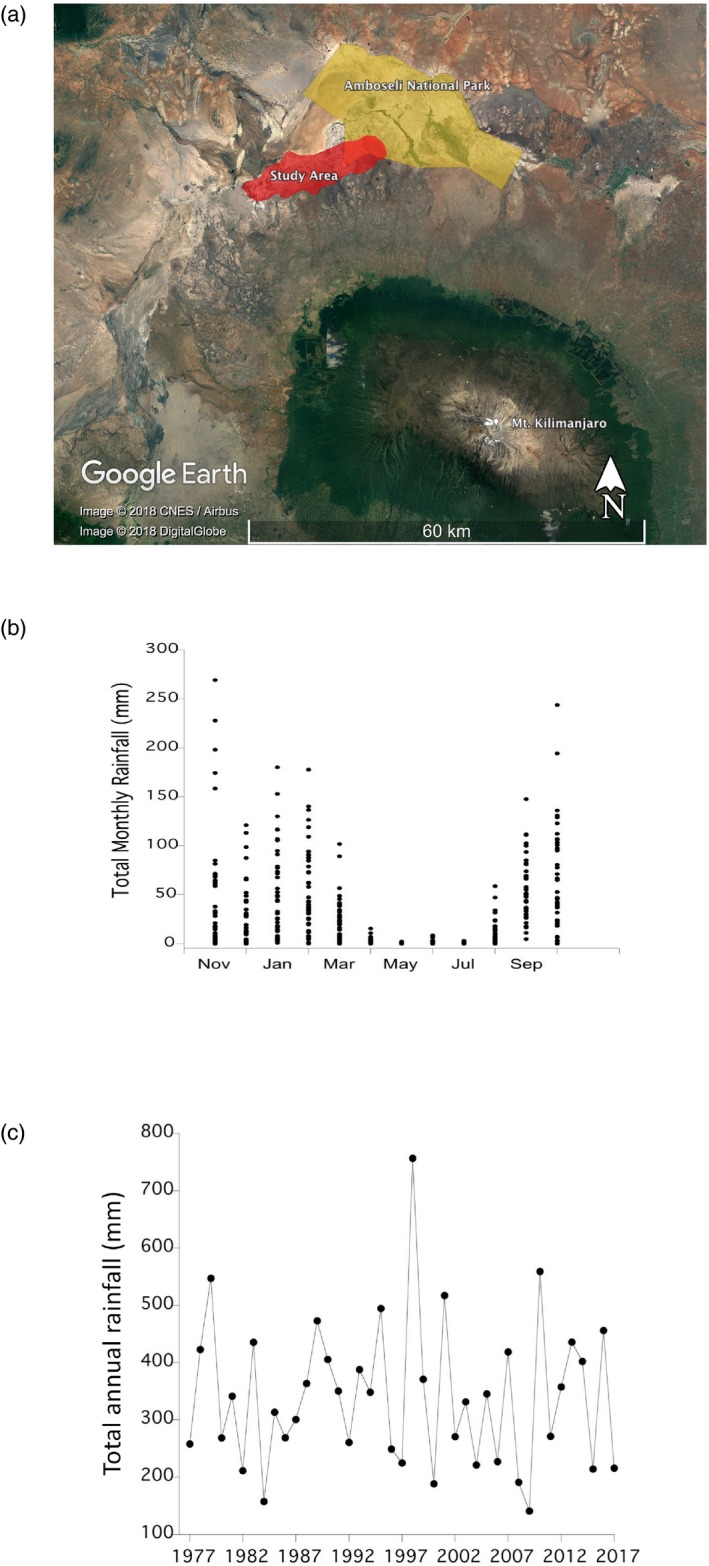
(a) Location of Amboseli National Park (yellow shading), the study are (red shading) and surrounding areas; image courtesy of Google Earth, C. Markham, and J. Gordon. (b) Total rainfall by month; open circles are individual monthly values (*n* = 499 months), showing variability across months and the predictable 5‐month dry season from June through October. (c) Total rainfall by hydrological year in Amboseli (November–October)

The Amboseli basin of southern Kenya—a large dry Pleistocene lake basin at the northern base of Mt. Kilimanjaro, bordering northern Tanzania and southern Kenya—is a place of extremes (Figure 1a). The intense heat of mid‐day can peak at 45°C during the hottest months; overnight lows during the coolest months can drop as low as 5°C. The basin has no drainage, and the soils are highly mineralized and alkaline (Stoessell & Hay, 1978; Trueman, Behrensmeyer, Tuross, & Weiner, 2004). Rain almost never falls between June and October, and is highly unpredictable between November and May, with annual totals averaging 350 mm (Figure 1b,c; Alberts et al., 2005; Western & Vanpraet, 1973). This combination of abiotic factors and the presence of a full complement of large and small herbivore grazers and browsers produces a dry savanna mosaic that consists of grasses, shrubs, scattered groves of *Acacia* woodland and large areas of bare ground.

Baboons are the most common nonhuman primate in the basin; vervet monkeys (*Chlorocebus pygerythrus*) and lesser galagos (*Galago senegalensis*) occur as well. Predators on baboons include lions, leopards (increasingly rare in recent decades), hyenas (*Crocuta crocuta*), pythons (*Python sebae*) and various large birds of prey (Altmann & Altmann, 1970). Cheetah (*Acinonyx jubatus*) and two species of jackal (*Canis mesomelas* and *C. aureus*) are also present in the basin but rarely or never take baboons as prey. The major human population in the basin is Maasai, a pastoralist group that occupies a large swath of southern Kenya and northern Tanzania. In recent years, the Maasai population in the Amboseli basin has grown, reflecting a common pattern for people living near National Parks in developing countries (Wittemyer, Elsen, Bean, Burton, & Brashares, 2008). This human population growth has resulted in habitat changes, including persistent over‐grazing by livestock (Groom & Western, 2013; Western, Groom, & Worden, 2009).

The baboon population in Amboseli, which is part of a larger, continuous baboon population in southern Kenya and northern Tanzania, lies in a hybrid zone between two species. The population is composed primarily of yellow baboons (*Papio cynocephalus*), but occasional immigration of anubis (or olive) baboons (*P. anubis*) into the basin has resulted in low to moderate levels of recent admixture in *c*. 30% of individuals in the study population (Alberts & Altmann, 2001; Charpentier et al., 2012; Samuels & Altmann, 1986; Tung, Charpentier, Garfield, Altmann, & Alberts, 2008; see also Wall et al., 2016 for a genomic analysis of historic admixture). This admixture is not particularly unusual for a baboon population: Baboon species are not highly specialized, either morphologically or ecologically, and freely hybridize at all known contact zones (Ackermann, Rogers, & Cheverud, 2006; Bergman, Phillips‐Conroy, & Jolly, 2008; Jolly, 1993; Tung, Charpentier, Mukherjee, Altmann, & Alberts, 2012; Tung et al., 2008; Wango et al., 2017; Winder, 2014).

The number of baboons in the Amboseli basin has risen and fallen over the decades, largely in response to changes in the availability of *Acacia* woodlands. Baboons in many habitats are dependent upon trees, for two reasons. First, baboons do not sleep on the ground: Throughout their nearly continental range, they sleep either on cliffs or in trees. Second, in Amboseli, *Acacia* trees represent a major food resource for baboons. Baboons eat the flowers, green seed pods, fresh and dry seeds, and gum exudate of both species of *Acacia*, spending a greater proportion of their time on the products of *A. xanthophloea* than on *A. tortilis* (Alberts et al., 2005; Post, 1982).

In the 1960s, when Jeanne and Stuart Altmann first arrived in Amboseli to study baboons, a decade before they established the long‐term Amboseli Baboon Research Project, *Acacia* woodlands were abundant and the baboon population was relatively dense, estimated at *c*. 73 baboons per km^2^ in the central part of the basin. Over the next several decades, the *Acacia* woodlands declined dramatically in this area, at least partly in response to the pressure of browsers that were increasingly protected in the central basin (Struhsaker, 1967, 1973, 1976; Western & Vanpraet, 1973). In response, the baboon population in the central basin also declined dramatically, from *c*. 2,500 baboons in the early 1960s to fewer than 200 baboons by the mid‐1980s, at a density of less than 2 baboons per km^2^ (Altmann, Hausfater, & Altmann, 1985; Samuels & Altmann, 1991). During this same period, vervet monkeys—the other diurnal primate species in Amboseli—also declined dramatically and nearly went locally extinct in some areas of Amboseli (Cheney, Seyfarth, Andelman, & Lee, 1988; Isbell, Cheney, & Seyfarth, 1990, 1991; Struhsaker, 1973, 1976).

Baboons avoided local extinction by shifting their home ranges out of the central part of the basin where tree loss was greatest. The groups studied by the Amboseli Baboon Research Project, for instance, moved *c*. 6 km towards the south‐western edge of the basin, where the *Acacia* woodlands were still relatively robust (Alberts et al., 2005; Bronikowski & Altmann, 1996). Today, the south‐western edge of the basin has the densest baboon population of anywhere in the basin. This concentration may be facilitated by the growing Maasai population in that area: Much of the baboon's drinking water during the 5‐month dry season, when rain pools are not available, comes from Maasai wells and watering holes. Overall, the baboon population in the Amboseli basin has undergone a period of modest growth during the past two decades, which it is still experiencing. While the population is far below the density it achieved during the 1950s and 1960s, it has expanded considerably relative to its low point in the mid‐1980s, now encompassing *c*. 1,000–1,200 baboons (S.C. Alberts, E.A. Archie, J. Altmann, J. Tung, unpublished data).

## 3 BABOON ECOLOGY AND FLEXIBILITY

The persistence and recovery of the Amboseli baboon population in the face of dramatic habitat change reflects the highly flexible and adaptable nature of baboons. Baboons (genus *Papio*) have a near‐continental distribution in Africa, occurring in a wide range of habitats that span deserts with less than 100 mm of rainfall annually to montane forests with as much as 2,000 mm of rainfall annually (e.g. Brain, 1990; Cheney et al., 2004; Hamilton, Buskirk, & Buskirk, 1976; Henzi, Byrne, & Whiten, 1992; Higham et al., 2009; Lodge, Ross, Ortmann, & MacLarnon, 2013). Baboon species all follow the same general foraging strategy: They eat primarily plant parts (leaves, fruits, grass blades, tree gum and plant underground storage organs) and employ a highly selective approach to identifying the most profitable plant parts to consume; they supplement this diet with invertebrates and occasionally take small vertebrates (Altmann, 1998; Byrne, Whiten, Henzi, & McCulloch, 1993; Hamilton, Buskirk, & Buskirk, 1978; Norton, Rhine, Wynn, & Wynn, 1987; Post, 1982; Whiten, Byrne, Barton, Waterman, & Henzi, 1991). Their ability to occupy such a breadth of habitat types is not achieved by extensive dietary or morphological specializations within the genus: No clear ecological separation was detectable among six well‐recognized baboon taxa in a study of eight measures of habitat variability, including vegetation, climate, and geological and soil features (Winder, 2014). Winder (2014) also found that habitat variation within species is generally greater than variation between species (see also Wango et al., 2017).

In addition to being highly successful generalist foragers, baboons are also highly social, and their sociality is almost certainly a key component of their ecological flexibility. In baboons, as in most mammals, males are the dispersing sex and females remain in their natal group throughout their lives (Pusey, 1987). Thus, females have the opportunity to form strong and stable social bonds with kin, as well as with other female group mates (Seyfarth, 1976; Seyfarth, Silk, & Cheney, 2014; Silk, Alberts, & Altmann, 2006; Silk, Altmann & Alberts 2006). However, females also form strong social bonds with adult males, which may persist for months or even years in some contexts (Seyfarth, 1978; Silk, Roberts, Barrett, Patterson, & Strum, 2017; Smuts, 1985). Baboons use these social relationships to manage intraspecific competition, confront predation risk, mitigate disease risk, manage psychosocial stress and gain information about the environment.

## 4 SURVIVAL DURING THE INFANT AND JUVENILE PERIOD

Baboons are born with eyes open, and they have the ability to cling and to suckle. Beyond these abilities, they are relatively helpless, requiring near‐constant contact with the mother to survive the first 6 months of life. Over the course of the nearly five‐decade study in Amboseli, mortality in the first year of life has averaged 23% ± 2% (*M* ± *SE*) and has ranged from 0% to 60% (calculated annually; *N* = 46 years and 1,299 live‐born infants). The average value falls in the range of average infant mortality for other wild nonhuman primate populations (Bronikowski et al., 2011; Colchero et al., 2016).

As a result of pre‐adult mortality—deaths between birth and sexual maturity—only *c*. 50% of live‐born females reach the median age of first birth (5.95 years) and *c*. 44% of live‐born males reach the median age of first mate guarding episode (7.7 years for males), the closest proxy that we can reliably measure for age at first reproduction for males (Alberts & Altmann, 2003; McLean, Altmann, Archie, & Alberts, in review). Mortality is highest during infancy, defined here as the first year of life. Age‐specific survival rates increase dramatically between the first and fourth years of life, and reach a maximum in the fourth year of life for females (nearly 97% survival for 3‐ to 4‐year‐old females) and the fifth year of life for males (nearly 96% survival for 4‐ to 5‐year‐old males) (Alberts & Altmann, 2003).

Causes of death are difficult to establish in Amboseli, because predators and scavengers rapidly consume corpses. Further, our ability to establish a cause of death depends on the cause itself. For instance, deaths that involve a sudden disappearance of an otherwise healthy infant (usually when observers are not present) are likely to involve predators. These account for a full 43% of infant deaths; in the majority of these cases, we have no direct evidence of the cause of death, although in some we have circumstantial evidence of predators (e.g. nervousness and alarm‐calling by the baboons when the infant is first observed missing, or wounds on the mother's arms). We have direct or corroborating evidence about cause of death for approximately one quarter infant deaths (i.e. 67 of 287 deaths that occurred in the first year of life, out of 1,299 live births). Of these, about one‐third were caused by disease or obvious pathologies, and about half were violent deaths—that is, the infants were known to be killed by predators or humans, or were killed by conspecifics (including male infanticide, during aggressive interactions between groups, or as a presumed result of female–female competition); a few died in accidents such a falling from trees or drowning at water sources. Because some causes of death are much more likely to be ascertained than others, these proportions certainly do not reflect an unbiased sample.

### 4.1 Positive social influences on immature survival

For an Amboseli baboon, surviving infancy and the juvenile period requires not only avoiding predators and disease, but also learning to identify and consume more than 250 types of food in order to successfully navigate the transition to nutritional independence (Altmann, 1998). The social environment—particularly but not only interactions with kin—represents an important source of information as well as protection against threats; at the same time, the social environment can pose significant dangers to the developing animal.

**Figure 2:**
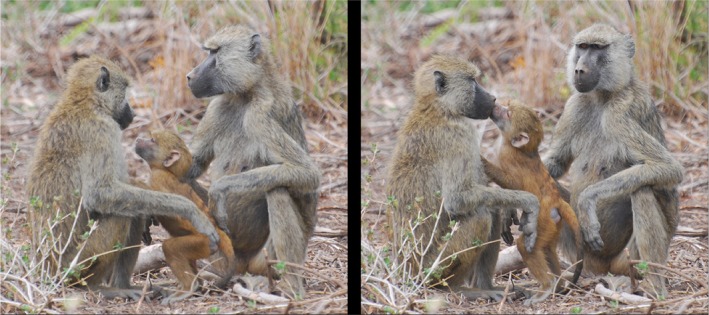
Muzzle‐sniffing by an infant baboon in Amboseli. Muzzle‐sniffing appears to be an important source of information about novel foods. Photo courtesy of Catherine Markham

Baboons take a highly selective approach to foraging. This means that infants must not only learn which species to eat, but which parts of each species. For example, with many grass species baboons specifically target meristem tissue (Altmann, 1998; see also Post, 1982; Whiten et al., 1991). Acquiring foraging skills therefore depends on the presence of tolerant and knowledgeable adults whom the developing animal can observe during foraging. In most primates, this role is primarily filled by the mother, but other animals can play a substantial role as well (Altmann, 1998; Coussi‐Korbel & Fragaszy, 1995; Janson & van Schaik, 1993; King, 1994). Evidence of active teaching of immature animals is absent in baboons as it is in most other nonhuman animals (King, 1994; Laland & Hoppitt, 2003; Thornton & Raihani, 2008). However, the baboon social environment is well suited to promote social learning, because the close proximity and frequent interactions between adults and immatures mean that infants will often be sitting in and around plants that their neighbours are actively consuming. Not only do older animals thus model the consumption of particular plant species and parts, they also tolerate infants that pick up dropped food scraps. In addition, infants frequently sniff the muzzles of other animals and disproportionately do so when others are eating (Figure 2; Altmann, 1998; King, 1994).

Mounting evidence supports the idea that the social environment can also help reduce the prevalence or richness of parasites and/or the fitness costs of parasite infection, through a variety of socially mediated process (Ezenwa, Ghai, McKay, & Williams, 2016; see also Rushmore, Bisanzio, & Gillespie, 2017). Evidence from several mammal species supports this idea. For example, in Yellowstone wolves, the mortality risks associated with infection by sarcoptic mange can be nearly or completely offset by living with pack‐mates rather than solitarily (Almberg et al., 2015). In baboons, too, disease risk may be offset by social behaviour. For instance, features of the gut microbiome can have functional consequences for the host, including resistance to invading pathogens (Dillon, Vennard, Buckling, & Charnley, 2005; Kamada, Chen, Inohara, & Nunez, 2013; Lozupone, Stombaugh, Gordon, Jansson, & Knight, 2012; Nie, Zhou, Qiao, & Chen, 2017), and in Amboseli baboons, social group membership and social network relationships predict both the taxonomic structure of the gut microbiome and the structure of genes encoded by gut microbial species (Grieneisen, Livermore, Alberts, Tung, & Archie, 2017; Tung, Barreiro, et al., 2015). Data from chimpanzees indicate that this social transmission of the gut microbiome may be beneficial, promoting species richness within individuals and creating a microbial meta‐community that preserves microbial diversity and that may enable individuals to recover from events that deplete their gut microbiome (Moeller et al., 2016).

**Figure 3:**
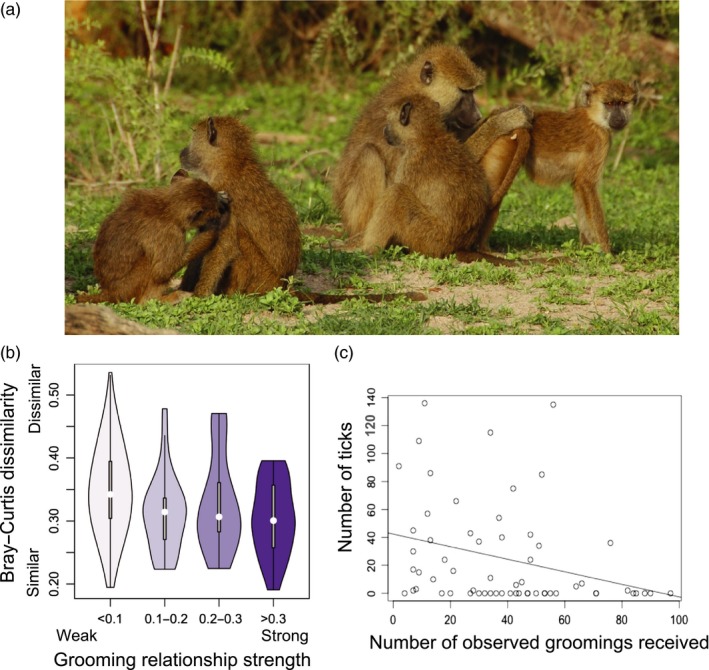
(a) An adult female baboon grooms a juvenile while other juveniles rest and groom nearby. (b) Gut microbiome dissilimarity, as measured by the Bray–Curtis dissimilarity index, is highest among pairs with the weakest grooming relationships. Details in Tung et al. (2015); figure modified from Tung et al. (2015). (c) Individuals that receive more grooming have fewer ticks. Y‐axis shows the count of ticks recovered from the entire body, while an individual baboon was anaesthetized for blood draw and morphometric measurements; x‐axis shows the total number of times an individual was observed being groomed in the 6 months prior to the tick count. Details in Akinyi et al. (2013); figure modified from Akinyi et al. (2013)

A common primate behaviour directly linked to both microbiome composition and to other aspects of health is social grooming; infant and young juvenile baboons are groomed intensively by mothers, siblings and unrelated juveniles and adults (Figure 3a). Importantly, close grooming partners in Amboseli share more similar gut microbiomes than individuals who do not groom each other (Figure 3b; Grieneisen et al., 2017). In addition, baboons that receive more grooming have lower tick loads than individuals that receive less grooming (Figure 3c), and high tick loads are associated with lower packed red cell volume in the blood in Amboseli (Akinyi et al., 2013). High tick loads can also present a direct threat to infant primate survival in some habitats (Brain, 1992; Brain & Bohrmann, 1992), indicating that the health benefits of grooming may have a significant effect on infant and juvenile survival.

### 4.2 Negative social influences on immature survival

Conspecifics can also pose serious threats to an infant's survival. Sexually selected infanticide is well documented in baboons, as in a number of other mammals and birds. In the Okavango delta of Botswana, sexually selected infanticide accounts for at least 38% and possibly up to 70% of baboon infant deaths (Cheney et al., 2004; Palombit, Seyfarth, & Cheney, 1997). In comparison, infanticide is relatively uncommon in Amboseli, possibly accounting for as little as 2.3% of infant deaths over all decades of the study (Zipple et al., 2017). However, the risk of infanticide varies with population demography in Amboseli. When the density of baboon social groups was relatively low in the 1970s and 1980s, so that males had fewer social groups to choose among when they were dispersing, infanticide accounted for at least 11% of infant deaths and was concentrated in groups where many dependent young were present (Zipple et al., 2017). Thus, infanticide is a contingent tactic among male baboons in Amboseli that occurs in response to particular demographic contexts. In these same demographic contexts, immigrant males may persistently target pregnant females in aggressive attacks, with females subsequently experiencing pregnancy termination (Alberts, Sapolsky, & Altmann, 1992; Pereira, 1983; Zipple et al., 2017).

Intergroup competition with conspecifics also represents a danger for immature baboons. Baboon groups have overlapping home ranges, and baboon social groups in Amboseli spend nearly a third of their time each month in areas of home range overlap with neighbouring baboon groups. As a result, each social group experiences intergroup encounters several times each month on average (Markham, Guttal, Alberts, & Altmann, 2013). While intergroup encounters most often involve one group displacing another with no physical conflict, they can occasionally escalate to a lethal level. In such circumstances, infants and young juveniles are the most common victims (Markham, Alberts, & Altmann, 2012; Shopland, 1982; S.C. Alberts, E.A. Archie, J. Altmann, J. Tung, unpublished data).

Within social groups, too, infants and young juveniles can be vulnerable to conspecifics other than infanticidal males. Specifically, the retrieval and carrying of young infants by nonfamily group members, usually adolescent females, can have harmful or even fatal consequences. Notably, in some species of primates and other mammals females “allomother” infants that are not their own, grooming them, carrying them during group travel and even, in some species, suckling them, conferring potential benefits on both mother and offspring (Fairbanks, 1990; Heldstab, van Schaik, & Isler, 2017; McKenna, 1979; Obrien & Robinson, 1991; Packer, Lewis, & Pusey, 1992; Perry, 1996; Stanford, 1992); similar benefits of helpers‐at‐the‐nest have long been recognized for birds (reviewed in Crick, 1992). However, in other species, including baboons, the taking and carrying of infants by nonparental individuals is more likely to represent competition among adult females (Altmann, 1980; Kohda, 1985; Quiatt, 1979; Shopland & Altmann, 1987; Silk, Rodman, & Samuels, 1979). At least 5% of infant deaths in Amboseli have occurred after a group member (usually an adolescent female) has taken an infant from the mother and carried or restrained it for hours or days. Baboon infants appear especially vulnerable to this risk in the first 2 months of life, when the infants themselves are unable to easily escape from the kidnapper; all but two of the confirmed kidnapping deaths have occurred in this period.

## 5 GROWTH AND DEVELOPMENT

Infant baboons weigh *c*. 0.71 kg at birth. In Amboseli, females grow at a rate of 4.9 g/day until *c*. 7 years of age (well past age at first birth), when both statural growth and mass gain slow and then cease within a year or two. Males in Amboseli grow at a rate of 5.5 g/day until *c*. 5 years of age, at which point they enter a growth spurt and gain 12.7 g/day until *c*. 8 years of age (approximately the age at first mate guarding; Altmann & Alberts, 2005). After age 8 years, both statural and mass growth appear to slow (for growth data from captive baboons see Druelle, Aerts, D'Aout, Moulin, & Berillon, 2017). Baboons subsisting entirely on wild foods in Amboseli may represent relatively slow‐growing animals; baboons are capable of substantially faster growth rates, both in the wild and in captivity (see, e.g. Druelle et al., 2017; Johnson, 2003).

### 5.1 Maternal and social influences on development

Offspring growth rates in baboons, as in many organisms, are influenced by maternal effects. For instance, in Amboseli, maternal dominance rank explains 23% of the variation in growth rates of both male and female offspring, with offspring of high‐ranking mothers being relatively large‐for‐age and offspring of low‐ranking mothers being relatively small‐for‐age (Altmann & Alberts, 2005; see also Johnson, 2003 for similar results in wild chacma baboons).

Importantly, this pattern of rank‐influenced growth may be strongly dependent upon the nature and abundance of the food source. In Amboseli, we see an effect of maternal rank only in wild‐feeding baboons that have no anthropogenic source of nutrition (our main study subjects). We conducted a parallel analysis in a social group that lives near a tourist lodge, where energetic resources are more easily obtainable in the form of food refuse. There, the effect of maternal dominance rank was not statistically significant, although it showed a trend in the same direction (Altmann & Alberts, 2005). This comparison highlights the fact that the social environment—specifically dominance rank—will be less important for growth in more energy‐rich environments, and conversely that in energy‐limited environments social behaviour is a major source of variation in resource acquisition.

Relatively rapid growth contributes to comparatively early sexual maturation. The offspring of high‐ranking females in Amboseli not only grow faster, they also reach sexual maturity earlier: In a group of 30 adult females, the daughter of the highest‐ranking female (ordinal rank 1) is likely to reach maturity *c*. 6 months earlier than the daughter of the lowest‐ranking female (Altmann & Alberts, 2003; see also Altmann & Alberts, 2005; Charpentier, Tung, et al., 2008; Onyango, Gesquiere, Altmann, & Alberts, 2013a). A similar pattern is reported in a number of other primates and some other mammals (Clancey & Byers, 2016; Holand et al., 2004; Pusey, 2012). However, age at maturity is influenced by a number of other social effects beyond maternal dominance rank, including some that are sex‐specific. For instance, the family environment affects age at maturity for females, but not males: Female maturity is accelerated when females have more maternal sisters present but delayed in groups with more adult females overall (Charpentier, Tung, et al., 2008). In contrast to females, male sexual maturity is slightly accelerated in groups with more adult females, implicating the presence of females as a developmental trigger for male development, but is unaffected by the presence of sisters (Charpentier, Tung, et al., 2008).

### 5.2 Paternal influences on development

Several studies have documented that young baboons receive beneficent attention from adult males in general, and from their biological fathers in particular. For instance, adult males in Amboseli commonly intervene in agonistic interactions that involve juveniles (invariably supporting the youngest animal in the interaction), and they do so disproportionately on behalf of their own offspring (Buchan, Alberts, Silk, & Altmann, 2003). Adult males in two different wild baboon populations are known to spend more time in proximity to their biological offspring than expected by chance, suggesting relatively active paternal investment in baboons (Huchard et al., 2013; Onyango, Gesquiere, Altmann, & Alberts, 2013b).

We and others have documented that this paternal presence has functional consequences for offspring in wild baboon populations. Early in the infant's life, the presence of the father can provide protection against infanticidal males and other potentially dangerous conspecifics (Moscovice et al., 2010; Nguyen, Van Horn, Alberts, & Altmann, 2009; Weingrill, 2000). During the juvenile period, the presence of the father enhances the development of social bonds among paternal half‐sisters (Lynch, Di Fiore, Lynch, & Palombit, 2017; see also Widdig, 2007). The presence of the father can also accelerate physical development: in Amboseli, individuals whose fathers are present for most of the juvenile period mature earlier than individuals whose fathers disperse to other social groups when the offspring is relatively young (Charpentier, Van Horn, Altmann, & Alberts, 2008). In addition, wild juvenile chacma baboons gain access to richer food patches by associating with their biological fathers (Huchard et al., 2013), supporting the idea that paternal presence has the potential to affect juvenile growth.

In the aggregate, then, young baboons may receive considerable support from fathers. Baboons are not the only nonmonogamous primate species in which direct or indirect influences of paternal presence have been documented (e.g. rhesus macaques: Widdig, Langos, & Kulik, 2016; Widdig, Nurnberg, Krawczak, Streich, & Bercovitch, 2001; chimpanzees: Murray, Stanton, Lonsdorf, Wroblewski, & Pusey, 2016; gorillas: Rosenbaum, Silk, & Stoinski, 2011; black howlers: Van Belle, Garber, Estrada, & Di Fiore, 2014; Assamese macaques: Minge, Berghanel, Schulke, & Ostner, 2016). However, paternal care has not, to my knowledge, been documented in other nonmonogamous mammals or birds. Under what circumstances is paternal care likely to evolve in multi‐male animal societies? Direct paternal care will be strongest when paternity certainty is high, when offspring greatly benefit from direct paternal care, and when the energetic and opportunity costs of paternal care are low. Baboons appear to meet all of these conditions in spite of the fact that females mate with multiple males, and that mating occurs year‐round. First, female sexual swellings provide relatively precise indicators of ovulation in baboons (but not in all primates: Nunn, 1999), and the paracallosal skin of females turns from black to red during the first trimester of the 6‐month gestation (Altmann, 1973; Bailey, Eberly, & Packer, 2015; Miller, Livermore, Alberts, Tung, & Archie, 2017). Thus, adult males have several cues about the timing of conception. Second, the direct benefits of paternal presence to young baboons, described above, may be partly attributable to the very large size of adult male baboons relative to all other members of the group, which may enable males to provide more care, at lower cost, than in primate species that are less size‐dimorphic. Third, if the direct paternal care that male baboons exhibit stems chiefly from their large size and the proximity that they and their offspring maintain with each other, paternal protection of youngsters during foraging and other activities may impose few or no energetic or opportunity costs to males who are also seeking mating opportunities.

### 5.3 Fitness implications of variation in growth and development

Does the variation in growth and development described in the previous sections have consequences for lifetime fitness? Timing of sexual maturation per se probably has little or no significance for fitness outcomes in the ecological context of Amboseli. Fitness can be strongly influenced by age at first reproduction in some demographic contexts (reviewed in Stearns, 1992), but the age at which individuals begin to reproduce in Amboseli contributes little to no variation in individual lifetime reproductive success (McLean et al., in review). This is because reproductive life span—the largest component of lifetime reproductive success for both males and females—is almost entirely determined by age at death, which is extremely variable in Amboseli as in most wild primate populations (Altmann, Altmann, & Hausfater, 1988; Colchero et al., 2016; McLean et al., in review). Thus, although variation in age at maturity appears substantial when considered in isolation, its variation is quite small in comparison with variation in age at death and this limits its potential impact on individual fitness in a population such as Amboseli (McLean et al., in review).

However, while the direct fitness consequences of delayed versus accelerated maturation may be limited for Amboseli baboons, delayed growth during the juvenile period has potentially important implications through other pathways. For one thing, baboons that grow more slowly may have lower juvenile survival; while such a survival disadvantage has not been examined in baboons, it has been observed in captive rhesus macaques, a species that has a generalist foraging strategy and slow life history similar to baboons (Nunez, Grote, Wechsler, Allen‐Blevins, & Hinde, 2015). In addition, baboons that grow more slowly may achieve a smaller adult body size: Baboons in Amboseli tend to be consistently small‐for‐age or large‐for‐age throughout the juvenile period, suggesting a lack of compensatory growth prior to reproduction (Altmann & Alberts, 2005). Indeed, poor early environments have been linked to smaller adult body size in many vertebrates, including humans (Festa‐Bianchet, Jorgenson, & Reale, 2000; Hoddinott et al., 2013; Lumey, Stein, & Susser, 2011; Pettorelli et al., 2002; Post, Stenseth, Langvatn, & Fromentin, 1997; Pucciarelli et al., 2000; Solberg, Garel, Heim, Grotan, & Saether, 2008; Toigo, Gaillard, & Michallet, 1999). And, while compensatory growth is documented in many animal taxa, including humans and other mammals (reviewed in Hector & Nakagawa, 2012; Mangel & Munch, 2005; Metcalfe & Monaghan, 2003), it does not occur in all species or all ecological contexts (e.g. Festa‐Bianchet et al., 2000; Solberg et al., 2008; Toigo et al., 1999). In addition, in small mammals, fish and lizards, compensatory growth can lead to significant fitness costs, most notably in reduced adult survival (reviewed in Hector & Nakagawa, 2012; Mangel & Munch, 2005; Metcalfe & Monaghan, 2003), although evidence for such costs is limited in large, slow‐growing mammals (see, e.g. Marcil‐Ferland, Festa‐Bianchet, Martin, & Pelletier, 2013).

The fitness consequences of small adult body size may be substantial. Although we know virtually nothing about this subject in wild primates, it is a key predictor of survival, fertility, or both, in a large range of mammals and birds (e.g. Soay sheep: Coltman et al., 1999; Wilson et al., 2007; bighorn sheep: Festa‐Bianchet et al., 2000; Gaillard, Festa‐Bianchet, Delorme, & Jorgenson, 2000; red deer: Post et al., 1997; roe deer: Gaillard et al., 2000; domestic sheep: Gunn, 1977; cliff swallows: Brown & Brown, 1998; great tits: Both, Visser, & Verboven, 1999). Indeed, positive selection for larger body size appears to be a highly consistent pattern in natural populations of both vertebrates and invertebrates, with larger size generally favoured by both natural and sexual selection (Kingsolver & Pfennig, 2004).

Thus, social effects on growth and maturation take on additional significance if the nutritional differences that produce slow juvenile growth result in small adult body size, and if adult body size is linked directly or indirectly to survival or reproduction in baboons. We currently lack the data to directly test these links in the Amboseli baboons, and I know of no other wild primate population with the requisite data. However, several lines of evidence from Amboseli support the idea that early nutrition has lasting consequences for phenotypic quality in baboons. The first line of evidence was established by Stuart Altmann (Altmann, 1991), who conducted a detailed study of nutritional intake by 12 individual baboons, 6 males and 6 females between 30 and 70 weeks of age (the period of transition to nutritional independence). He found that none of the subjects came close to achieving their estimated optimal energy intake for growth and maintenance (given constraints of time, other macronutrients and so on; see Altmann, 1991, 1998 for details). However, when he examined the lifetime reproductive output of the females in his dataset, 15 years later when they had lived through much or all of their adulthood, he found that their estimated energy shortfall during infancy strongly predicted multiple measures of lifetime reproductive success. For instance, the degree of energy shortfall during the weaning period accounted for 89% of the variance in female reproductive life span and 95% of the variance in the number of live infant females produced over the lifetime. In other words, infant females that took in more energy during the transition to independence had higher lifetime fitness (Altmann, 1991). Several other, less direct lines of evidence about the importance of early nutrition and growth are discussed below.

## 6 ADULTHOOD: COMPETITION AND COOPERATION

Social living enhances the competitive and defensive power of an individual in multiple ways. It facilitates collective action (against predators and against conspecific groups), reduces individual predation risk and enhances the acquisition and transfer of information about the environment (e.g. Alexander, 1974; Chapman & Chapman, 2001; Markham & Gesquiere, 2017; Powers & Lehmann, 2017). However, social living also ensures that competition with group mates is a defining feature of life (Janson & van Schaik, 1988). Baboon ecology is certainly influenced by competition, and in some cases, commensalism, with other animal species in their environment (Altmann & Altmann, 1970), but they experience the most intense competition with conspecifics. The resources over which baboons compete include food (primarily plants), waterholes, sleeping sites and sexual partners; competition for nonsexual social partners may also be important but has been less extensively studied than other types of competition.

A second defining feature of baboon social life is cooperative affiliation. Cooperation occurs both in the form of collective action (e.g. displacing of other social groups during intergroup encounters, aggressive coalitions during within‐group conflicts, collective defence against predators) and by providing social services to conspecifics (e.g. support during agonistic interactions and grooming). Individual baboons vary in their frequency of competitive and cooperative interactions, and also presumably in their effectiveness in these interactions. This variation provides a rich vein for research on variation in fitness outcomes. Below, I first discuss competitive and cooperative interactions that are known or suspected to influence fitness outcomes, and then summarize our research on sources of individual differences in these outcomes, including differences that arise in early life.

### 6.1 Within‐group competition and cooperation: Social dominance rank and social affiliation

In baboons, as in many social species, competition with group mates is strongly influenced by individual dominance rank. The relationship between dominance rank and male mating success, for instance, has been intensively studied in primates and other mammals (reviewed in Cowlishaw & Dunbar, 1991 for primates, Clutton‐Brock, 2016 for mammals). In Amboseli, high‐ranking male baboons experience higher mating success than low‐ranking males, but the magnitude of their advantage is density‐dependent: The strongest advantage to rank occurs in relatively small social groups, in which high‐ranking males are able to enforce a mating queue. For instance, the highest‐ranking adult male in a small group (e.g. 3 adult males) can expect to obtain up to 85% of mating opportunities, while the highest‐ranking adult male in a group with 10 adult males is likely to obtain less than 20% of mating opportunities, assuming a relatively constant sex ratio across group sizes (Alberts, Watts, & Altmann, 2003). In larger social groups, agonistic coalitions between males allow lower‐ranking males to “jump the queue” and obtain more mating opportunities than predicted by their dominance rank (Alberts et al., 2003).

Dominance rank also influences access to food. While direct estimates of food intake are difficult to obtain in wild animals, high‐ranking animals are known to obtain more nutrients than lower‐ranking ones in a wide range of primates and other mammal species (e.g. Hanuman langurs: Koenig, 2000; baboons: Barton, Byrne, & Whiten, 1996; capuchin monkeys: Di Bitetti & Janson, 2001; bison: Vervaecke, Roden, & De Vries, 2005; reindeer: Holand et al., 2004; red deer: Ceacero et al., 2012; Wilson et al., 2011). How does dominance rank influence nutritional intake? For one thing, high‐ranking individuals have priority of access to food and other resources. For instance, high‐ranking baboons of both sexes in Amboseli experience fewer feeding interruptions from conspecifics (terminations of feeding bouts brought about by the approach and/or aggressive behaviour of a conspecific), and hence longer feeding bouts than lower‐ranking individuals (Post, Hausfater, & McCuskey, 1980).

In addition, high‐ranking individuals may intensify this advantage through direct interference with group mates during foraging. Many baboon foods (e.g. grass corms, tree gum, tubers) require digging, opening, extracting or separating from a substrate (Altmann, 1998; Shopland, 1987), suggesting that these types of foods may be particularly good targets for the conspecific feeding interruptions that are common in baboon groups. Surprisingly, however, these interruptions are independent of a food's dispersion, its processing time and its rate of yield: higher‐ranking baboons interrupt the feeding of lower‐ranking ones indiscriminately with respect to food type (Shopland, 1987). Notably, in contrast to the interrupting animal, the response of the victim is highly sensitive to both processing time and the rate of yield of a food item: Baboons are more likely to resist attempted takeovers of foods that require more processing time or have a higher yield rate. In combination, these results suggest that aggressors do not interrupt feeding bouts strategically to maximize their own nutrient gain, but instead to disrupt and harass lower‐ranking conspecifics, even over dispersed food sources (Shopland, 1987). The consequence is an intricately mixed pattern of scramble and contest competition over food within social groups (Markham & Gesquiere, 2017), producing advantages to high‐ranking animals even when food resources are dispersed.

The energetic advantages to high rank that result from this competitive regime probably underlie the higher reproductive rates that high‐ranking female baboons experience. Specifically, high‐ranking females have shorter periods of post‐partum (lactational) amenorrhoea following the birth of an infant: A difference of 10 rank positions corresponds to a 33‐day difference in the duration of post‐partum amenorrhoea (Gesquiere, Altmann, & Alberts, 2018). Similar results are reported in other baboon populations (Johnson, 2003; Packer, Collins, Sindimwo, & Goodall, 1995; Smuts & Nicolson, 1989; Wasser, Norton, Kleindorfer, & Rhine, 2004; Wasser, Norton, Rhine, Klein, & Kleindorfer, 1998; see also Setchell & Wickings, 2004 in mandrils). Further, both female baboons and women in nonindustrial societies typically experience a negative energy balance during lactation (i.e. they expend more calories than they consume) and do not resume reproductive cycling until their infant has achieved sufficient nutritional independence that the mother returns to a positive energy balance (i.e. takes in more energy than she expends) (Ellison, 2003, 2017; Gesquiere et al., 2018; Valeggia & Ellison, 2009; see also Emery Thompson, 2013 for chimpanzees). In baboons, social dominance rank appears to strongly influence the ability of females to achieve this positive energy balance (Gesquiere et al., 2018).

Baboon social life is also strongly influenced by affiliative social interactions, most notably the exchange of grooming. Female baboons in Amboseli spend 5‐15% of their time in grooming and other social activities, and grooming time increases when food is more abundant (i.e. in the wet season and in higher quality habitats: Alberts et al., 2005; Bronikowski & Altmann, 1996). In primates, grooming is exchanged both for being groomed and for rank‐related benefits, particularly agonistic support, in a wide range of primate species (see, e.g. meta‐analyses in Schino, 2007; Schino & Aureli, 2008). A well‐established model proposes that lower‐ranking females obtain rank‐related benefits in exchange for grooming higher‐ranking females, and thus that, in the aggregate, more grooming will be directed from lower‐ to higher‐ranking females than the reverse, and that grooming will be most frequent between adjacently ranked females (Seyfarth, 1977). These predictions are generally borne out in both New World and Old World primates (Schino, 2001; Tiddi, Aureli, & Schino, 2012). At the same time, female primates appear quite sensitive to reciprocity in the exchange of grooming: Female primates tend to preferentially groom those group mates from whom they receive the most grooming, and in baboons, the most stable social relationships are characterized by high reciprocity in grooming exchanges (Schino & Aureli, 2008; Silk, Alberts, et al., 2006; Silk et al., 2010a).

**Figure 4:**
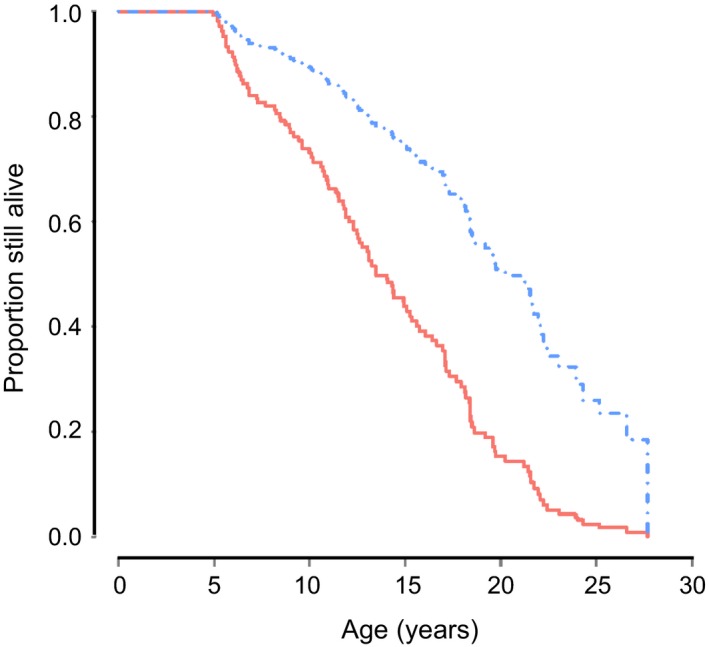
Survivorship for female baboons in Amboseli at the 75th percentile (blue) and 25th percentile (red) of social connectedness to other adult males and other adult females (effects combined; *N* = 204 females, Cox proportional hazards). Females at the 75th percentile for connectedness to other adult females were 34% less likely to die in a given year than females at the 25th percentile, and females at the 75th percentile for connectedness to adult males were 45% less likely to die in a given year than females at the 25th percentile. Modified from Archie et al. (2014)

In baboons, affiliative social interactions either directly cause variation in immature and adult survival or are predicted by other traits that do so. That is, the extent to which an adult female is socially integrated versus socially isolated within the context of the social group—measured in several different ways—has been linked to offspring survival, adult survival or both, in three different wild baboon populations, including Amboseli baboons (Figure 4; Archie, Tung, Clark, Altmann, & Alberts, 2014; McFarland et al., 2017; Silk, Alberts, & Altmann, 2003; Silk et al., 2009, 2010b). Further, the magnitude of this effect is quite striking: In Amboseli, adult females at the 75th percentile for social connectedness (a measure of the grooming frequency with social partners) live an average of 5 or more years longer than females at the 25th percentile of social connectedness (Figure 4 and Archie et al., 2014). This result for adult survival closely parallels studies in humans that demonstrate a link between social integration and both health and survival in adulthood (Holt‐Lunstad, Smith, & Layton, 2010; Marmot, 2004). Whether this effect is causal—so that social relationships in adulthood can mitigate the negative effects of other environmental variables on health—or whether social relationships are predicted by overall phenotypic quality, which in turns predicts survival, remains to be tested.

### 6.2 Between‐group competition: Group size

Competition between groups for access to rich food patches, water sources and sleeping sites can modify the net costs of competition within groups, depending upon group size. In general, larger groups should be more successful than smaller groups in between‐group competition, through their ability to displace smaller groups at resources. Thus, the disadvantages of within‐group competition, which will generally increase with increasing group size, may be offset by the advantages experienced by large groups in between‐group competition (Chapman & Chapman, 2001; Chapman & Valenta, 2015; Grove, 2012; Markham & Gesquiere, 2017; Powers & Lehmann, 2017; Scarry, 2013; van Schaik, 1983; Wrangham, 1980). This competitive tension suggests that intermediate‐sized groups may experience the most favourable balance of within‐group and between‐group competition.

**Figure 5:**
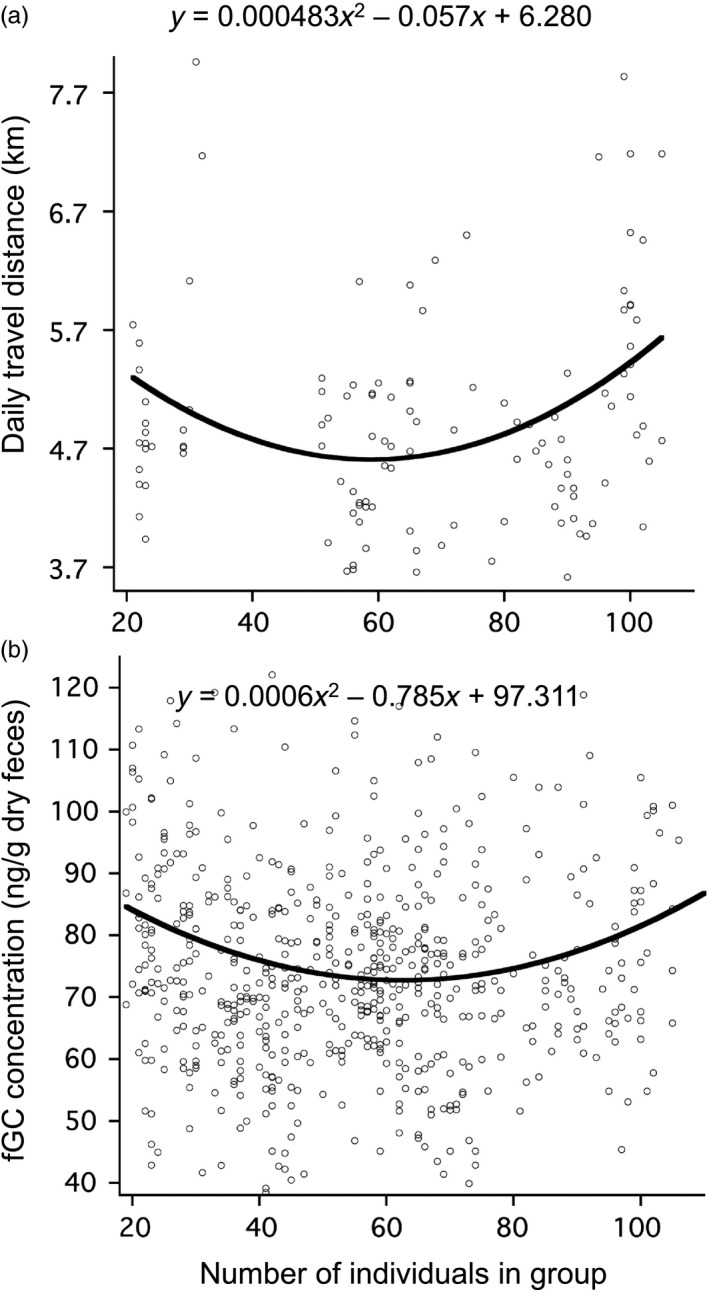
(a) Female baboons in the largest and smallest social groups travel the longest distances each day. (b) They also have the highest faecal glucocorticoid (fGC) concentrations. The figures show simple bivariate relationships, with the equations and lines describing the curvilinear relationships. Each point represents a single group‐month: *N* = 120 for records of daily travel, *N* = 632 for fGC. Modified from Markham et al. (2015)

In support, female baboons in Amboseli that live in intermediate‐sized groups have energetic advantages over females in large and small groups (Markham, Gesquiere, Alberts, & Altmann, 2015). We have demonstrated U‐shaped relationships between group size and (a) home range area, (b) average daily distance travelled by groups and (c) average glucocorticoid concentrations for females, with females in intermediate‐sized groups showing the lowest values for all three measures (Figure 5). These results are consistent with the idea (which awaits direct testing) that large groups are the most highly constrained by within‐group competition, whereas small groups are the mostly highly constrained by between‐group competition and predation pressures (Markham & Gesquiere, 2017; Markham et al., 2015).

## 7 THE LONG REACH OF EARLY LIFE

### 7.1 The effects of the early life environment on adult behaviour and fitness outcomes

As noted above, only *c*. 50% of females and *c*. 44% of males in Amboseli reach their sex‐specific age at first reproduction; thus, simply failing to reproduce is a major source of variation in individual fitness for Amboseli baboons, as for many organisms. However, even among baboons that reach adulthood, considerable variation in lifetime fitness is evident (Alberts, Buchan, & Altmann, 2006; Altmann et al., 1988; McLean et al., in review). While many sources of this variation remain unclear, it is increasingly obvious that in Amboseli, circumstances early in development (during infancy and the juvenile period) play a major role.

For instance, a detailed analysis of female fertility demonstrated that adult female baboons are less likely to resume sexual cycling and less likely to conceive during drought years than in years with average rainfall (Lea, Altmann, Alberts, & Tung, 2015). Notably, this effect is strongest among females that were themselves born during droughts. This result implicates early nutrition as a predictor of compromised fertility in adulthood (Lea et al., 2015). Similar “silver spoon” effects of early life condition on adult fertility have been reported in several other studies of birds and mammals (e.g. roe deer: Douhard et al., 2014; humans: Hayward, Rickard, & Lummaa, 2013; red deer: Nussey, Kruuk, Morris, & Clutton‐Brock, 2007; bighorn sheep: Pigeon & Pelletier, 2018; barn swallows: Balbontin & Moller, 2015; tawny owls: Millon, Petty, Little, & Lambin, 2011; house wrens: Bowers, Thompson, & Sakaluk, 2017; goshawks: Herfindal, van de Pol, Nielsen, Saether, & Moller, 2015).

**Figure 6:**
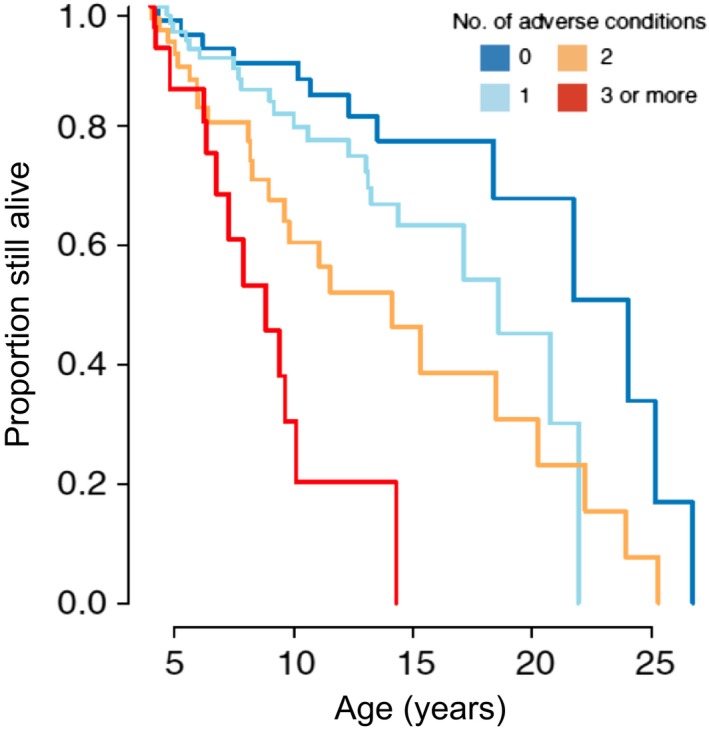
Survivorship for adult female baboons that experience different numbers of adverse circumstances in early life: blue line = no early adverse circumstances, red line = 3 or more early adverse circumstances (*N* = 196, hazard ratio = 1.9, Cox proportional hazards). Modified from Tung et al. (2016)

Early life circumstances predict not only fertility, but also adult survival in Amboseli females. In the case of survival, we examined six adverse circumstances during infancy and the juvenile period: drought in the first year of life, being born in a large group, being born to a low‐ranking mother, being born to a socially isolated mother, having a close‐in‐age younger sibling (i.e. a sibling born after an interbirth interval of 1.5 years or less, representing the lowest quartile of interbirth intervals) and losing one's mother before the age of 4 years (Tung, Archie, Altmann, & Alberts, 2016). Most of these circumstances have clear consequences for nutrition and resource acquisition; some or all may also function through psychosocial mechanisms. We observed a powerful negative effect of the accumulation of multiple early adverse circumstances on adult life span. Among individuals that survive to adulthood, females who experience ≥3 sources of early adversity die a median of 10 years earlier than females who experience one or no adverse circumstances; this is a large effect given that the median life span is 18.5 years among females that survive to adulthood (Figure 6).

Several other studies of birds and mammals have shown the effects of early life conditions on adult survival, although such studies typically focus on one or two measures of the early life environment, such as rainfall, temperature or population density (e.g. goshawks: Herfindal et al., 2015; red‐billed choughs: Reid, Bignal, Bignal, McCracken, & Monaghan, 2003; Mauritius kestrels: Cartwright, Nicoll, Jones, Tatayah, & Norris, 2014; oystercatchers: Van de Pol, Bruinzeel, Heg, Van der Jeugd, & Verhulst, 2006; humans: Hayward et al., 2013). In designing our study, we followed the tradition in the human literature and created a cumulative index that summed the number of adverse circumstances an individual experienced in early life. Such cumulative indices have proven highly predictive of a range of measures of health and survival in contemporary human populations (e.g. Evans, 2003; Felitti et al., 1998; Schilling, Aseltine, & Gore, 2008; Seeman, Epel, Gruenewald, Karlamangla, & McEwen, 2010). Our cumulative model explained slightly more variance than a multivariate model that entered all six adverse events separately. Furthermore, a cumulative model that excluded maternal loss and close‐in‐age sibling—the two early adverse circumstances that reliably predicted adult survival in the multivariate model—still explained significant variation in adult survival. This result indicates that the accumulation of multiple adverse circumstances in early life, even those that may appear to have relatively little impact on their own, has negative consequences for long‐term survival (Tung et al., 2016).

Intriguingly, females who experience the most adverse circumstances in early life also tend to be socially isolated in adulthood from other adult females, although not from adult males (Tung et al., 2016). Because adult social relationships are linked to health and/or survival in several species of mammals, including humans (Archie et al., 2014; Barocas, Ilany, Koren, Kam, & Geffen, 2011; Holt‐Lunstad et al., 2010; Silk et al., 2010b; Yee, Cavigelli, Delgado, & McClintock, 2008), this result suggests that social processes may partially explain the link between early adversity and adult survival. As noted above, it remains to be tested whether this link is causal, or whether social relationships are simply correlated with overall phenotypic quality, which in turn drives survival. If the link is causal, then the effects of early adversity on survival in Amboseli females may stem not only from nutrition‐based differences in female quality but may be partly explained by the relative social isolation of high‐adversity females.

Another feature of the relationship between the social environment and fitness outcomes that we have yet to fully understand is the link between the social integration of a female's *mother* during the female's early life and her survival as an adult (Tung et al., 2016). Of the six adverse circumstances that we examined, this one seems the least likely to be linked to early nutrition, again suggesting that social processes may have effects on survival that are not strictly linked to nutrition. In any case, the striking link between adult survival and social integration—both a female's own during adulthood, and her mother's during the female's early life—points to individual‐specific social experiences as potentially critical components of the environment that must be taken into account in understanding environmental influences on fitness.

### 7.2 Cohort effects versus individual‐specific effects

Cohort effects—effects of a common early environment on long‐term survival or reproduction—occur when sets of individuals are born close in time, in the same environment, and experience the same environmental conditions (e.g. rainfall or population density). In seasonal breeders, cohort effects and early life circumstances are often equivalent. For instance, for roe deer, year‐to‐year variation in rainfall may affect fawn survival, adult female body mass, adult female fertility and/or adult female survival, depending on the overall quality of the environment (Douhard et al., 2014; Gaillard et al., 1997). In red deer on the Isle of Rum, environmental quality is heavily driven by variation in population density; therefore, a striking cohort effect involves faster reproductive ageing for female red deer born in years with high population density (Nussey et al., 2007). In bighorn sheep, too, high population density in early life has negative consequences for both age at first reproduction and life span (Pigeon & Pelletier, 2018). Examples of such cohort effects on survival or reproduction, for one sex or both, are common in seasonally breeding animals, including lizards (Le Galliard, Marquis, & Massot, 2010; Marquis, Massot, & Le Galliard, 2008), red squirrels (Descamps, Boutin, McAdam, Berteaux, & Gaillard, 2009), and multiple species of birds (e.g. great tits: Visser & Verboven, 1999; Wilkin & Sheldon, 2009; barn owls: Roulin, 2002; kittiwakes: Cam, Monnat, & Hines, 2003; oystercatchers: Van de Pol et al., 2006).

In contrast, cohort effects will be greatly diminished—and potentially swamped by individual‐level variation—in species that are both highly social and nonseasonal. The first reason for this is that in nonseasonal breeders, births are spread throughout the year, so that individuals born within the same hydrological year (i.e. during the period between the beginning of the rains 1 year and the beginning of the rains the next) will often experience dramatically different environmental conditions in early life (e.g. if one is born in the dry season and one in the wet season, or one at the beginning of the year and one at the end). In addition, the period of maternal care, including lactation, may vary considerably for nonseasonal breeders: In female baboons in Amboseli, the period of lactational amenorrhoea between two successive live births ranges from 71 to 635 days (Gesquiere et al., 2018). These circumstances mean that, to capture early life effects, individual‐specific metrics of the early environment must be used, such as rainfall in the 12 months following an individual's birthdate (e.g. Lea et al., 2015), or the length of the birth interval following an individual's birth (e.g. Tung et al., 2016).

The second reason is that for primates the social environment may be profoundly different for different individuals, even if they are born in the same group at the same time. For instance, dominance rank and affiliative social relationships are two aspects of the environment that can have major consequences for individuals in many species. For baboons, both of these aspects of the social environment vary greatly both among individuals and within individuals over time (e.g. Archie et al., 2014; Lea, Learn, Theus, Altmann, & Alberts, 2014; Samuels, Silk, & Altmann, 1987; Silk, Alberts, et al., 2006; Silk, Altmann, et al., 2006). Such individual‐specific circumstances may amplify or diminish the effects of poor environmental conditions (Hamel, Gaillard, Festa‐Bianchet, & Cote, 2009; Lomnicki, 1978). For primates and other highly social species, individual differences in the environment may be greater in number or magnitude than aspects that are experienced in common. As a consequence, we may observe more environmentally driven individual‐level variation in highly social, nonseasonally breeding species such as baboons than in many other animals.

## 8 CONCLUSIONS: THE IMPORTANCE OF LONG‐TERM STUDIES

The synthesis of research presented here demonstrates some of the ways in which social interactions fundamentally shape the lifetime performance of animals. Many of these insights would be impossible to achieve without long‐term data. Most of the results described here—the effects of the social environment on growth and development, the link between the social environment and the survival of infants and adults, and the effects of early life circumstances on adult life span and fertility—required multiyear longitudinal data on known individuals. Equally importantly, the accumulation of long‐term behavioural and life‐history data has been accompanied, in Amboseli as in several other long‐term studies, by the accumulation of biological samples from known individuals. This sample collection has positioned us to begin probing the evolution of important traits by combining genetic, phenotypic and environmental data on the same individuals (e.g. see Lea, Altmann, Alberts, & Tung, 2016; Tung, Alberts, & Wray, 2010; Tung, Zhou, Alberts, Stephens, & Gilad, 2015; Wall et al., 2016; for examples of evolutionary genetic insights from other long‐term studies, see Abzhanov et al., 2006; Berenos et al., 2015; Johnston, Berenos, Slate, & Pemberton, 2016; Johnston et al., 2013; Rands et al., 2013).

Long‐term studies face limitations and challenges, just as short‐term studies do (Festa‐Bianchet, Douhard, Gaillard, & Pelletier, 2017). In spite of these challenges, which include the need for ongoing funding and the constant challenge of sustaining day‐to‐day data collection, the centrality of long‐term studies in the effort to understand the evolution of social behaviour and life histories is well recognized. Such studies are just beginning to realize their potential for providing unprecedented insight into the evolution and functional significance of social behaviour (Clutton‐Brock & Sheldon, 2010; Festa‐Bianchet et al., 2017).

## Data Availability

This study does not use data.
